# Supplementary figures and images for: Separable roles for RNAi in regulation of transposable elements and viability in the fission yeast Schizosaccharomyces japonicus
Source: PLoS Genet. 2022 Feb 28;18(2):e1010100. doi: 10.1371/journal.pgen.1010100 (PMC8912903; doi:10.1371/journal.pgen.1010100)

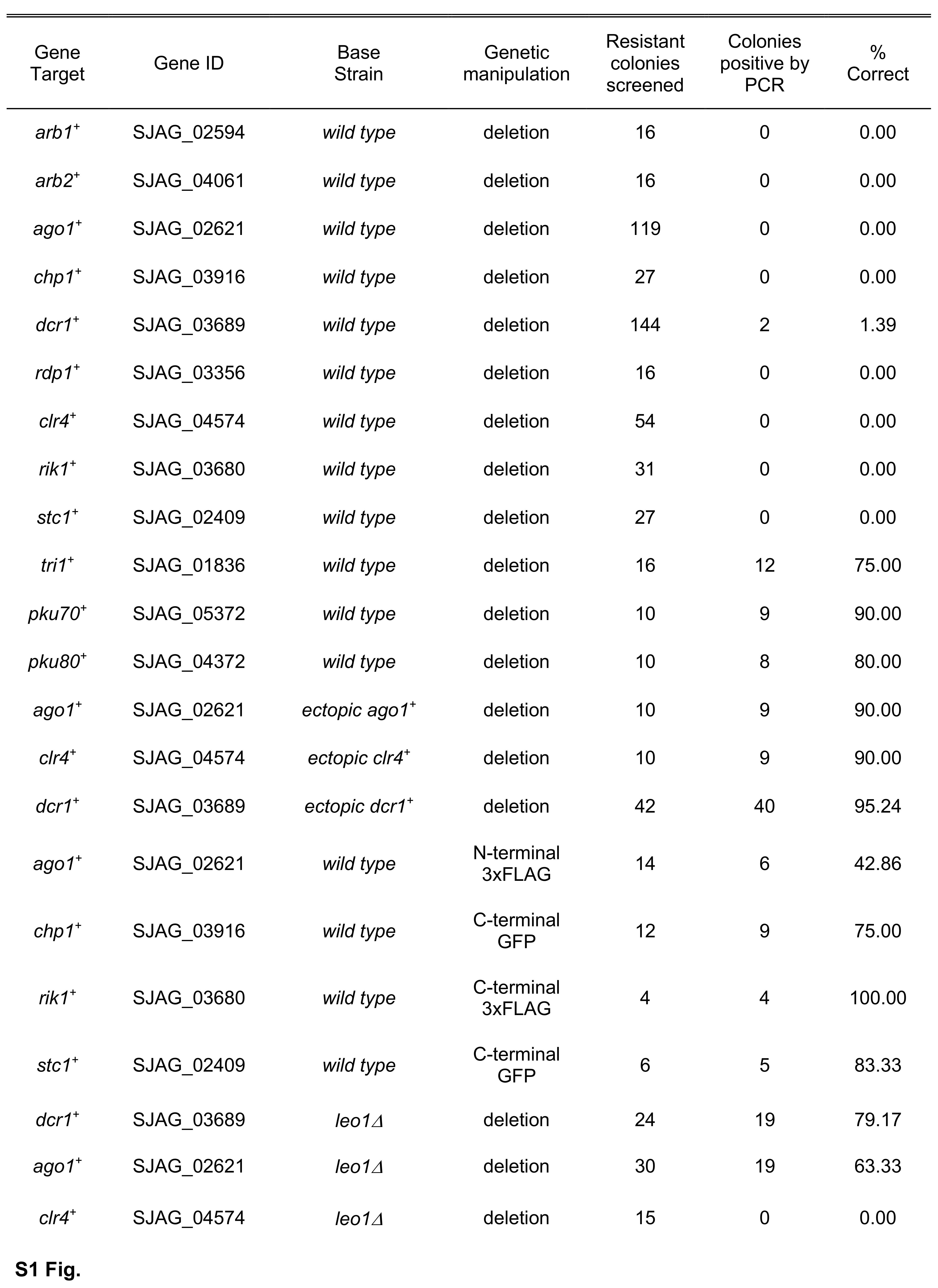

Supplement: S1 Fig — Knockout and tagging rates of targeted genes in the wild-type background, in the presence of an ectopic gene copy, or in a leo1Δ background. (TIF) [file pgen.1010100.s001.tif]

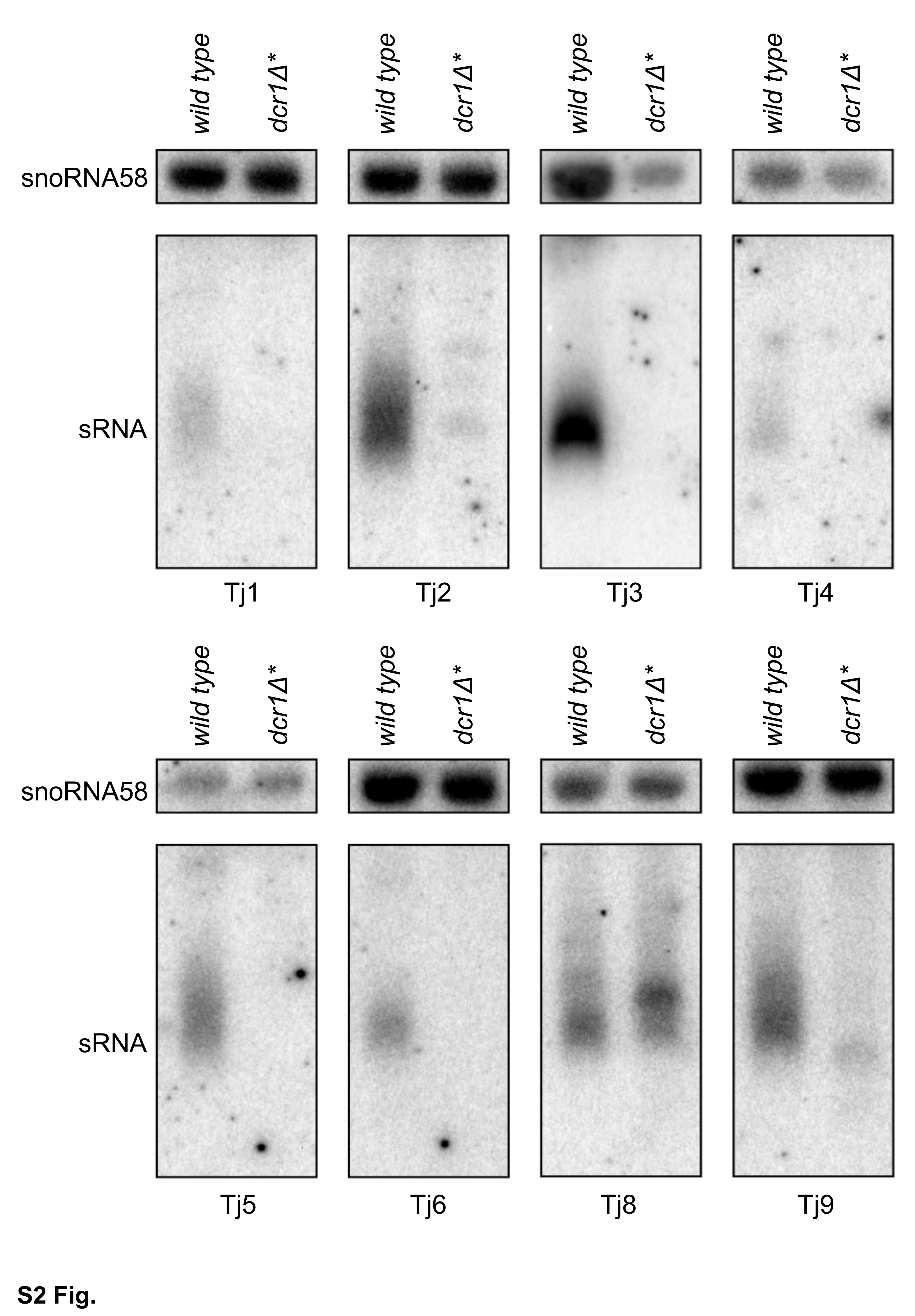

Supplement: S2 Fig — Northern blot of small RNA species isolated from wild-type and dcr1Δ* strains, probed with 32P end-labelled oligonucleotides, antisense to the indicated retrotransposon or snoRNA58, a loading control. (TIF) [file pgen.1010100.s002.tif]

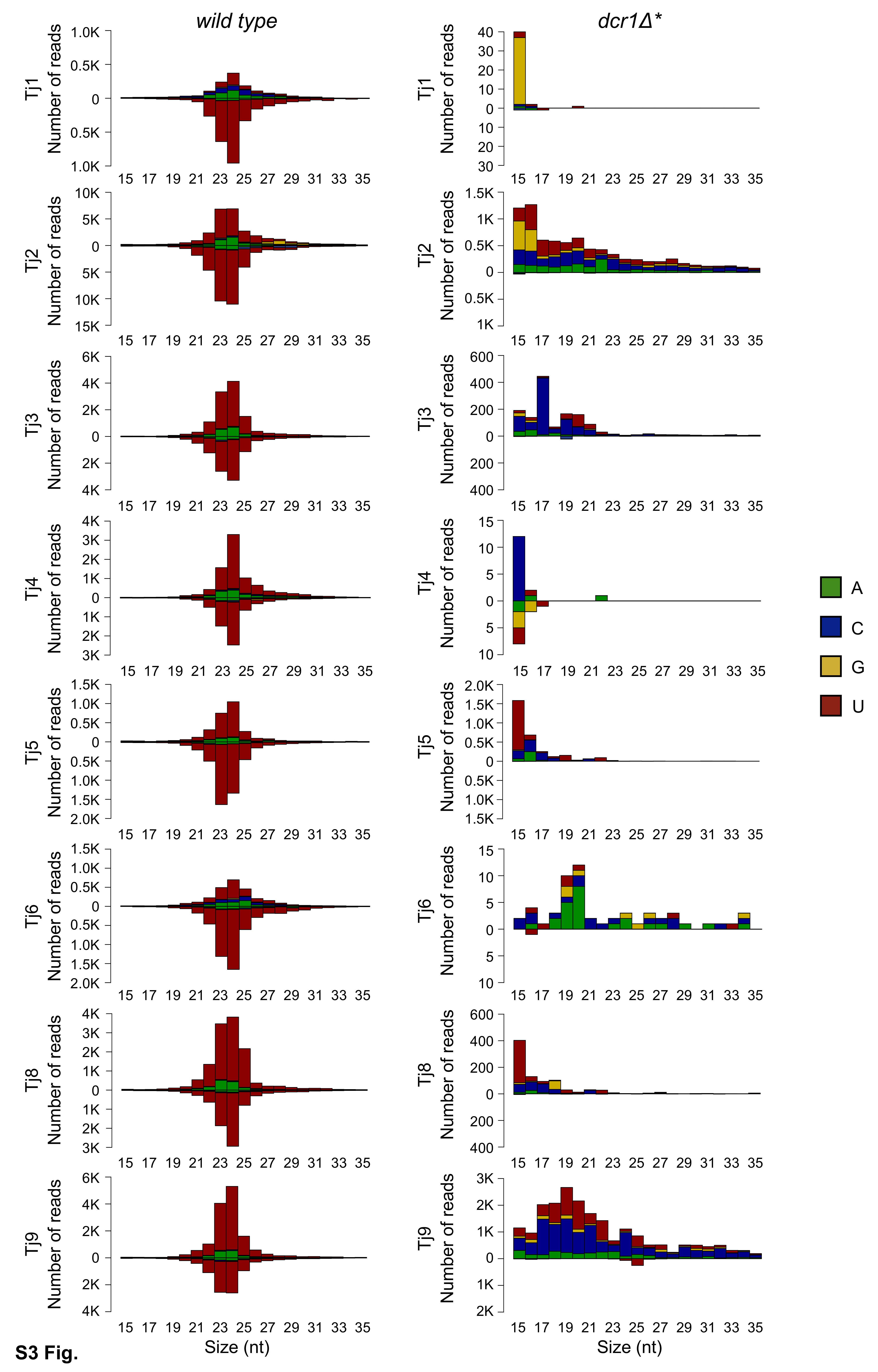

Supplement: S3 Fig — Size profile, strand bias and 5’ nucleotide preference of small RNA species that map to indicated retrotransposons, isolated from wild-type and dcr1Δ* strains. RNAs derived from the sense strand are plotted above the axis, whilst RNAs derived from the antisense strand are plotted below. (TIF) [file pgen.1010100.s003.tif]

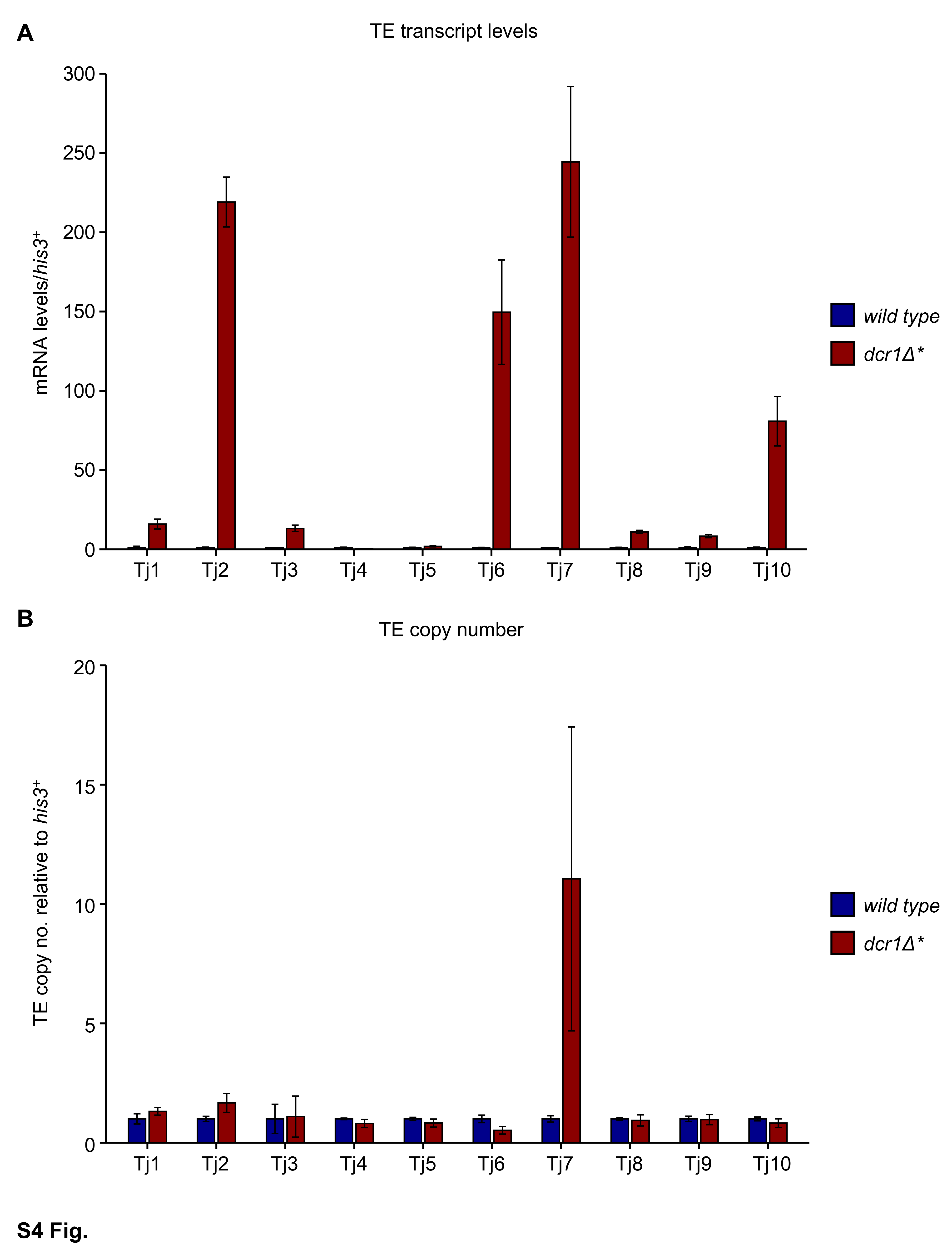

Supplement: S4 Fig — (A) RT-qPCR analysis of retrotransposon transcript levels, relative to his3+, normalised to wild-type. Data plotted are the mean ± SD from three replicates. Data for Tj7 and Tj10 are the same as in Fig 2C. (B) qPCR analysis of retrotransposon copy number, relative to his3+, normalised to wild-type. Data plotted are the mean ± SD from three replicates. Data for Tj7 and Tj10 are the same as in Fig 2D. (TIF) [file pgen.1010100.s004.tif]

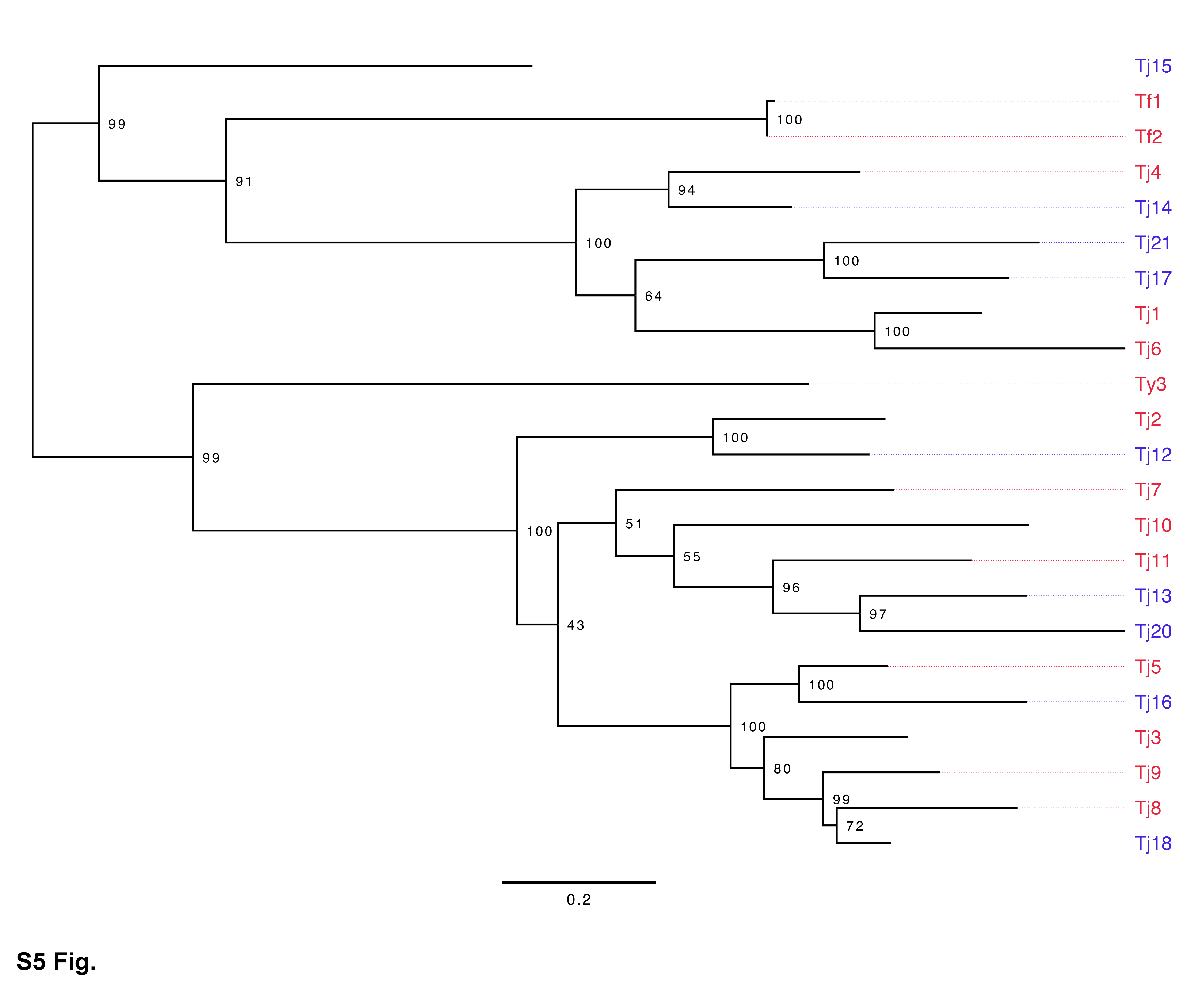

Supplement: S5 Fig — For each newly discovered retrotransposon, the sequences of the reverse transcriptase, RNase and integrase domains were aligned to the corresponding domains from Tj1-10, as well as S. pombe Tf1 and Tf2 and S. cerevisiae Ty3 using ClustalW (Tj19 was excluded as we could find only partial elements in which these domains were not present). IQtree2 was then used to build a single tree based on the three domains, with 1000 ultrafast bootstraps. IQtree2 selected TVM+F+R3 as the best-fitting base substitution model using the Bayesian Information Criterion (BIC). Node labels indicate bootstrapping values. Scale bar is in units of base substitutions per site. Retrotransposons discovered in this study (Tj12 –Tj21) are highlighted in blue, previously discovered retrotransposons (Tj1 –Tj11) are highlighted in red. (TIF) [file pgen.1010100.s005.tif]

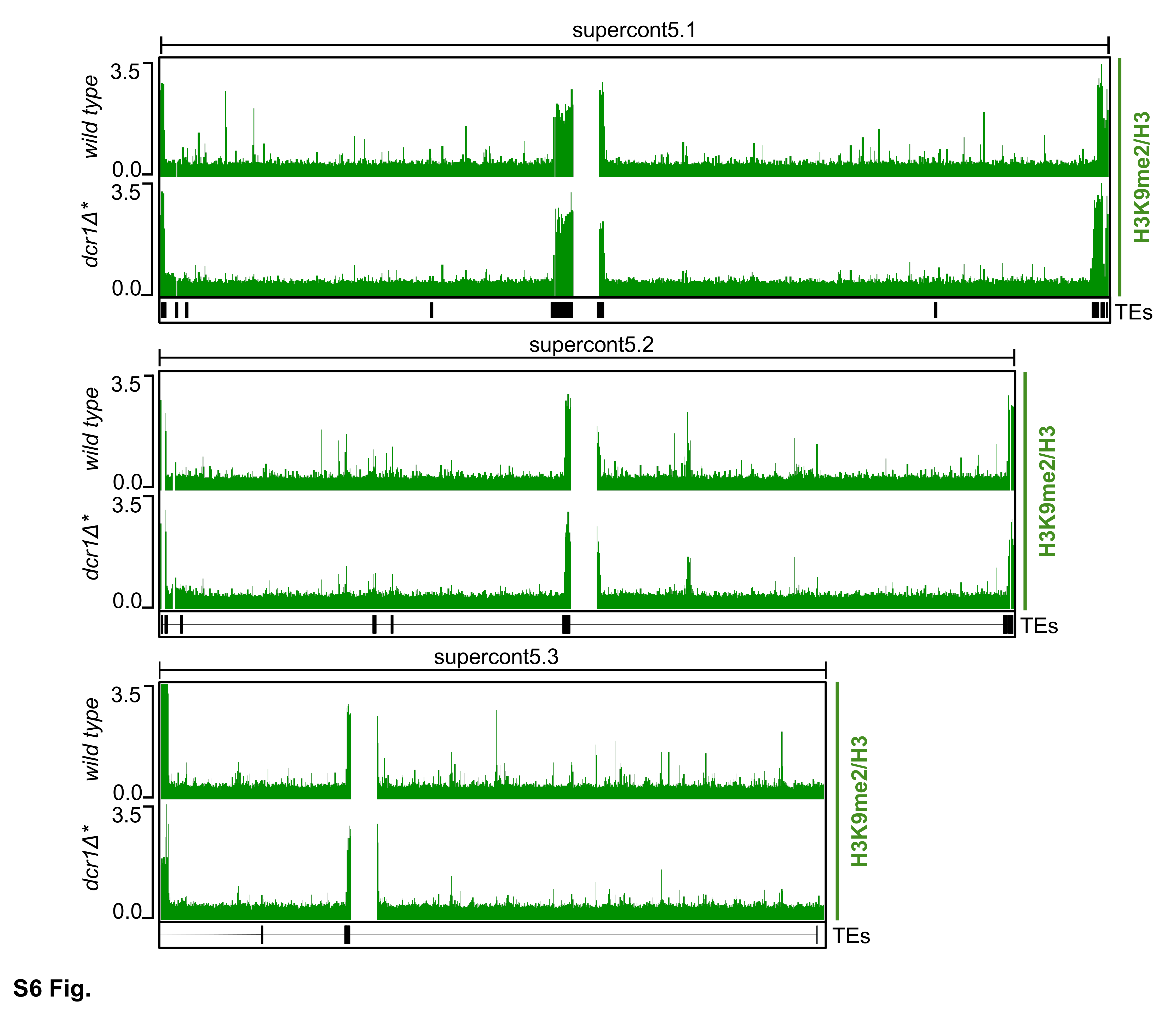

Supplement: S6 Fig — ChIP-seq profile of H3K9me2 in dcr1Δ* vs wild-type cells for the three main chromosomal contigs of the SJ5 annotation. Enrichments are given in reads per kilobase million (RPKM) and represent the ratio of input normalised anti-H3K9me2 precipitated chromatin over input normalised anti-H3 precipitated chromatin. TE locations are indicated by black bars underneath each data track. (TIF) [file pgen.1010100.s006.tif]

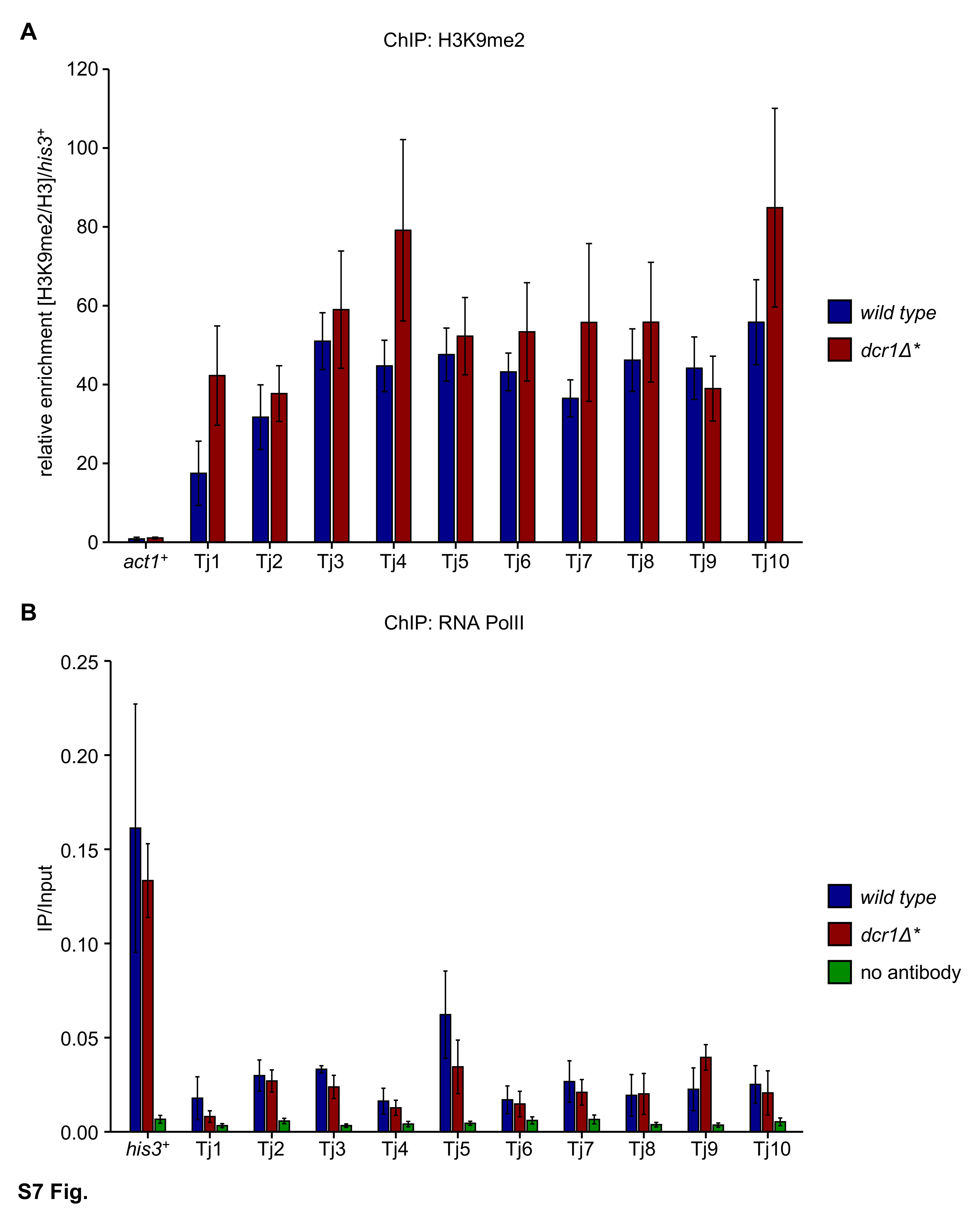

Supplement: S7 Fig — (A) ChIP-qPCR analysis of H3K9me2/H3 levels across retrotransposons, normalised to his3+. Data plotted are the mean ± SD from three replicates. (B) ChIP-qPCR analysis of RNA PolII levels across retrotransposons. his3+ is included to illustrate RNA PolII occupancy at a transcribed gene. Data plotted are the mean ± SD from three replicates. (TIF) [file pgen.1010100.s007.tif]

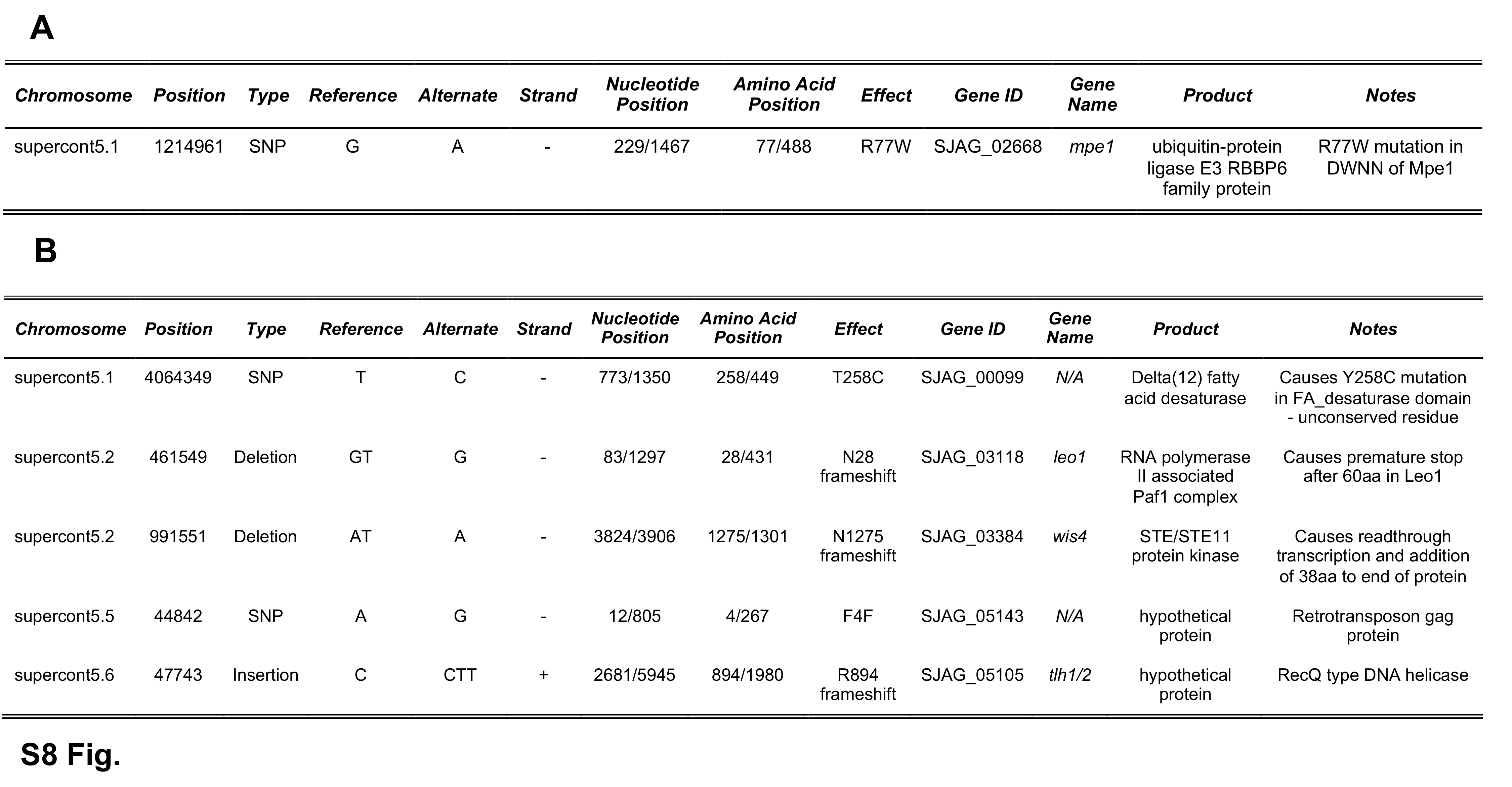

Supplement: S8 Fig — (A) Table of SNPs within coding regions sequenced from the dcr1Δ* strain. (B) Table of SNPs within coding regions sequenced from the dcr1Δ† strain. (TIF) [file pgen.1010100.s008.tif]

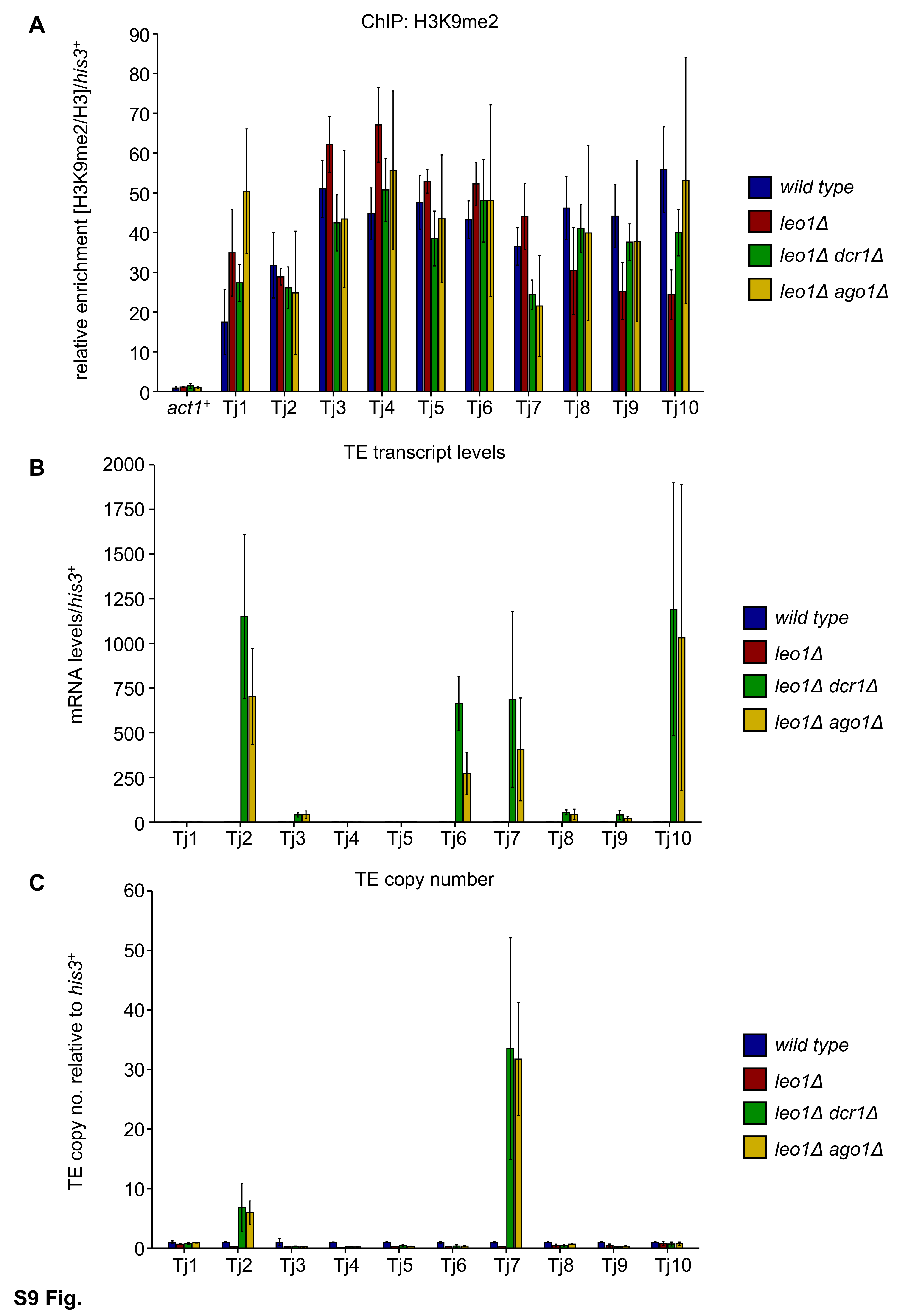

Supplement: S9 Fig — (A) ChIP-qPCR analysis of H3K9me2/H3 levels across retrotransposons, normalised to his3+. Data plotted are the mean ± SD from three replicates. (B) RT-qPCR analysis of retrotransposon transcript levels, relative to his3+, normalised to wild-type. Data plotted are the mean ± SD from three replicates. Data for Tj7 and Tj10 are the same as in Fig 4D & 4E. (C) qPCR analysis of retrotransposon copy number, relative to his3+, normalised to wild-type. Data plotted are the mean ± SD from three replicates. Data for Tj7 and Tj10 are the same as in Fig 4F. (TIF) [file pgen.1010100.s009.tif]

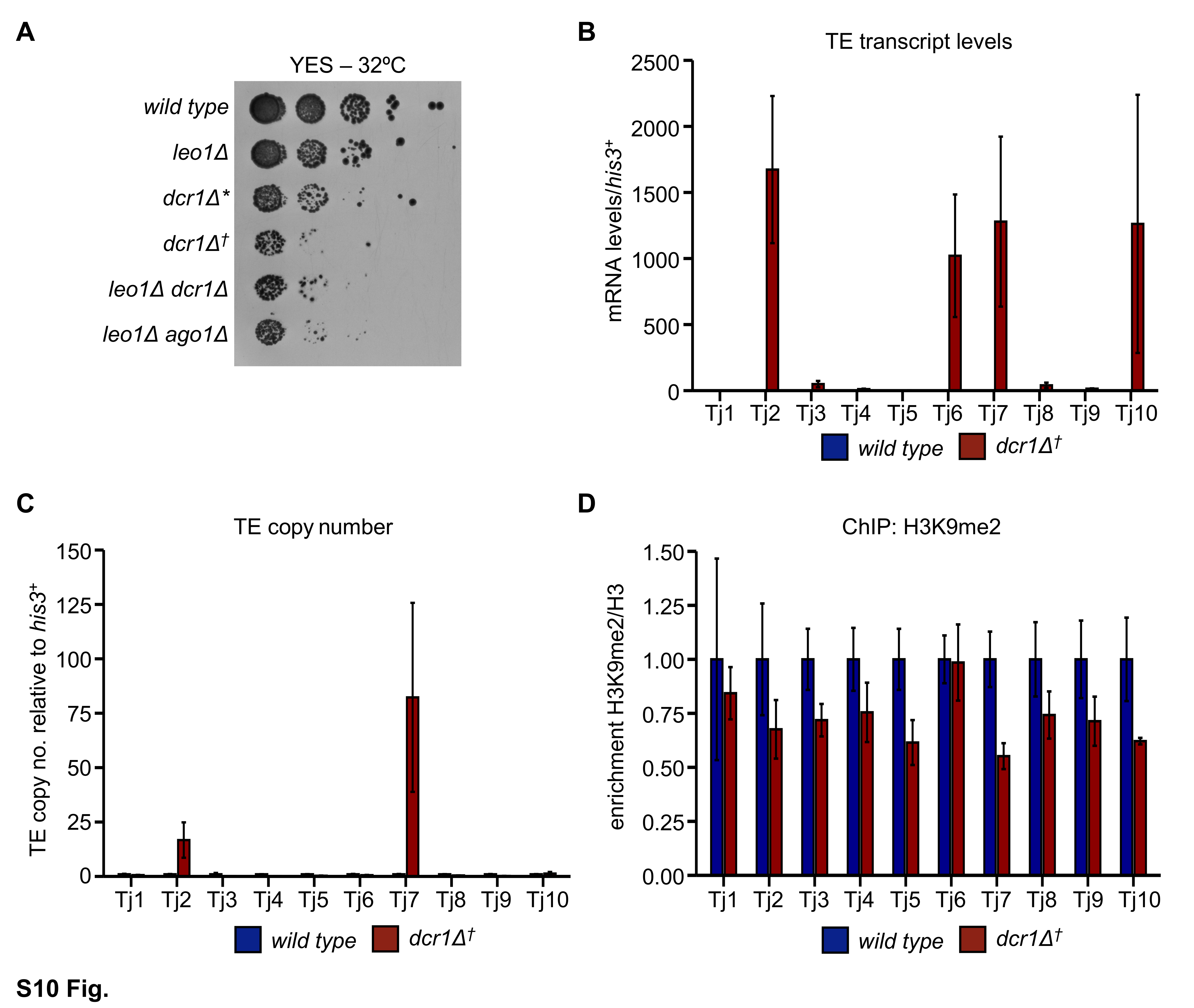

Supplement: S10 Fig — (A) Growth assay showing the growth phenotypes of the rare dcr1Δ survivor strains and the leo1Δ dcr1Δ and leo1Δ ago1Δ strains, in comparison to wild-type and leo1Δ strains. (B) RT-qPCR analysis of retrotransposon transcript levels, relative to his3+, normalised to wild-type. Data plotted are the mean ± SD from three replicates. (C) qPCR analysis of retrotransposon copy number, relative to his3+, normalised to wild-type. Data plotted are the mean ± SD from three replicates. (D) ChIP-qPCR analysis of H3K9me2/H3 levels across retrotransposons, normalised to wild-type. Data plotted are the mean ± SD from three replicates. (TIF) [file pgen.1010100.s010.tif]
