# Supplementary material for: Separable roles for RNAi in regulation of transposable elements and viability in the fission yeast Schizosaccharomyces japonicus
Source: PLoS Genet. 2022 Feb 28;18(2):e1010100. doi: 10.1371/journal.pgen.1010100 (PMC8912903; doi:10.1371/journal.pgen.1010100)
Supplement: S3 Table — (DOCX) [file pgen.1010100.s013.docx]

**S3 Table. *S. japonicus* strains used in this study.**

| **Genotype** | **Source** | **Strain Identifier** |
| --- | --- | --- |
| *h^−^ mat-P2028 ura4-D3 ade6(sj)-domE* | Niki Lab [1] | EHB2742 (NIG5384) |
| *h^+^ mat-2017 ura4-D3 ade6(sj)-domE* | Niki Lab [2] | EHB2743 (NIG5386) |
| *h^−^ mat-P2028 dcr1∆** (*dcr1^dis^::natMX6 mpe1^R77W^)*  *ura4-D3 ade6(sj)-domE* | This Study | EHB3054 |
| *h^−^ mat-P2028 dcr1∆^†^* (*dcr1^del^::natMX6 leo1^V61Stop^)*  *ura4-D3 ade6(sj)-domE* | This Study | EHB7518 |
| *h^−^ mat-P2028 tri1::ura4 ura4-D3 ade6(sj)-domE* | This Study | EHB3369 |
| *h^+^ mat-2017 tri1::ura4 ura4-D3 ade6(sj)-domE* | This Study | EHB3371 |
| *h^−^ mat-P2028 pku70::ura4 ura4-D3 ade6(sj)-domE* | This Study | EHB3254 |
| *h^+^ mat-2017 pku70::ura4 ura4-D3 ade6(sj)-domE* | This Study | EHB3256 |
| *h^−^ mat-P2028 pku80::ura4 ura4-D3 ade6(sj)-domE* | This Study | EHB3258 |
| *h^+^ mat-2017 pku80::ura4 ura4-D3 ade6(sj)-domE* | This Study | EHB3260 |
| *h^−^ mat-P2028 nmt1::ago1 ago1::NatMX6 ura4-D3 ade6(sj)-domE* | This Study | EHB4652 |
| *h^+^ mat-2017 nmt1::clr4 clr4::NatMX6 ura4-D3 ade6(sj)-domE* | This Study | EHB4732 |
| *h^+^ mat-2017 nmt1::dcr1 dcr1^dis^::NatMX6 ura4-D3 ade6(sj)-domE* | This Study | EHB5591 |
| *h^+^ mat-2017 nmt1::dcr1 dcr1^del^::NatMX6 ura4-D3 ade6(sj)-domE* | This Study | EHB5597 |
| *h^−^ mat-P2028 kanMX6-P3nmt1-3FLAG-ago1*  *ura4-D3 ade6(sj)-domE* | This Study | EHB4279 |
| *h^+^ mat-2017 kanMX6-P3nmt1-3FLAG-ago1*  *ura4-D3 ade6(sj)-domE* | This Study | EHB4282 |
| *h^+^ mat-2017 chp1-GFP-ura4 ura4-D3*  *ade6(sj)-domE* | This Study | EHB2668 |
| *h^+^ mat-2017 rik1-FLAG-NatMX6 ura4-D3*  *ade6(sj)-domE* | This Study | EHB2666 |
| *h^+^ mat-2017 stc1-GFP-ura4 ura4-D3*  *ade6(sj)-domE* | This Study | EHB2670 |
| *h^+^ mat-2017 leo1::ura4 ura4-D3 ade6(sj)-domE* | This Study | EHB5736 |
| *h^+^ mat-2017 leo1::ura4 dcr1^del^::NatMX6 ura4-D3 ade6(sj)-domE* | This Study | EHB7526 |
| *h^+^ mat-2017 leo1::ura4 ago1::NatMX6 ura4-D3 ade6(sj)-domE* | This Study | EHB7549 |

**References**

1. Furuya K, Niki H. Isolation of heterothallic haploid and auxotrophic mutants of *Schizosaccharomyces japonicus*. Yeast. 2009;26. doi:10.1002/yea.1662

2. Furuya K, Niki H. Construction of diploid zygotes by interallelic complementation of *ade6* in *Schizosaccharomyces japonicus*. Yeast. 2011;28. doi:10.1002/yea.1898
