# Supplementary material for: Separable roles for RNAi in regulation of transposable elements and viability in the fission yeast Schizosaccharomyces japonicus
Source: PLoS Genet. 2022 Feb 28;18(2):e1010100. doi: 10.1371/journal.pgen.1010100 (PMC8912903; doi:10.1371/journal.pgen.1010100)
Supplement: S4 Table — (DOCX) [file pgen.1010100.s014.docx]

**S4 Table. Plasmids used in this study**

| **Plasmid** | **Source** | **Plasmid Identifier** |
| --- | --- | --- |
| pFA6a-natMX6 | Carr Lab [1] | Euroscarf plasmid #P30437 |
| pFA6a-*ago1Δ-natMX6* | This Study | N/A |
| pFA6a-*clr4Δ-natMX6* | This Study | N/A |
| pFA6a-*dcr1Δ^dis^-natMX6* | This Study | N/A |
| pFA6a-*dcr1Δ^del^-natMX6* | This Study | N/A |
| pREP1 | Maundrell Lab [2] | N/A |
| pREP1SJ | This Study | N/A |
| pREP1SJ-*ago1^+^* | This Study | N/A |
| pREP1SJ-*clr4^+^* | This Study | N/A |
| pREP1SJ-*dcr1^+^* | This Study | N/A |
| pSO729 | Oliferenko Lab [3] | N/A |
| pSO729-*chp1^+^*-*GFP-ura4^sj+^* | This Study | N/A |
| pSO729-stc*1^+^*- GFP-*ura4^sj+^* | This Study | N/A |
| pFA6a-5FLAG-*natMX6* | Noguchi Lab [4] | Addgene plasmid #19343 |
| pFA6a-*rik1^+^*-5FLAG-*natMX6* | This Study | N/A |
| pFA6a-*kanMX6*-P3nmt1-3FLAG | Noguchi Lab [4] | Addgene plasmid #19336 |

**References**

1. Hentges P, van Driessche B, Tafforeau L, Vandenhaute J, Carr AM. Three novel antibiotic marker cassettes for gene disruption and marker switching in *Schizosaccharomyces pombe*. Yeast. 2005;22. doi:10.1002/yea.1291

2. Maundrell K. Thiamine-repressible expression vectors pREP and pRIP for fission yeast. Gene. 1993;123. doi:10.1016/0378-1119(93)90551-D

3. Pieper GH, Sprenger S, Teis D, Oliferenko S. ESCRT-III/Vps4 Controls Heterochromatin-Nuclear Envelope Attachments. Developmental Cell. 2020;53. doi:10.1016/j.devcel.2020.01.028

4. Noguchi C, Garabedian M v., Malik M, Noguchi E. A vector system for genomic FLAG epitope-tagging in *Schizosaccharomyces pombe*. Biotechnology Journal. 2008;3. doi:10.1002/biot.200800140
