# Supplementary material for: Separable roles for RNAi in regulation of transposable elements and viability in the fission yeast Schizosaccharomyces japonicus
Source: PLoS Genet. 2022 Feb 28;18(2):e1010100. doi: 10.1371/journal.pgen.1010100 (PMC8912903; doi:10.1371/journal.pgen.1010100)
Supplement: S5 Table — (DOCX) [file pgen.1010100.s015.docx]

**S5 Table. Oligonucleotides used in this study**

| **Oligonucleotide Name** | **Oligonucleotide Sequence** | **Usage** |
| --- | --- | --- |
| q_Tj1_F | TATTATTGTGCCGAGCGAAGG | qPCR |
| q_Tj1_R | GATGACCACCGTTCACTGAG | qPCR |
| q_Tj2_F | GGAGACAATCCCGTTGGCTA | qPCR |
| q_Tj2_R | ACTGGATGTCCGGGTCAAAC | qPCR |
| q_Tj3_F | AACCGTCCACATCGCCGTAT | qPCR |
| q_Tj3_R | CGGTCCAGCAACTCGTCAAT | qPCR |
| q_Tj4_F | GGTACCACCAGAGGGTGAAC | qPCR |
| q_Tj5_F | GCAGTGTTCAAGCGTCTTCGG | qPCR |
| q_Tj5_R | GGAAGTTGACGCGCTTCCTG | qPCR |
| q_Tj6_F | GAGCAAAGGAGTGGCAGAGT | qPCR |
| q_Tj6_R | TTCGTCTGCGGTCTGTAACC | qPCR |
| q_Tj7_F | AAGAACCGATACGCGCTGC | qPCR |
| q_Tj7_R | CTTGCTGAAGACGCGTGC | qPCR |
| q_Tj8_F | AGCCGTCGAATTCGCCCTA | qPCR |
| q_Tj8_R | TTGAGGCCGCGGTAGTCAAT | qPCR |
| q_Tj9_F | AACGGGCGATGAGGAAACAA | qPCR |
| q_Tj9_R | TTCCGTCGCTGAGGTCTTCC | qPCR |
| q_Tj10_F | CATTCGGACTCACCAATGCG | qPCR |
| q_Tj10_R | TGTTGATCGCGGTCCTTTGA | qPCR |
| q_his3_F | TACAGACCCTAAAATTGCCC | qPCR |
| q_his3_R | CAAGCTTGGACGTAGGATTA | qPCR |
| q_act1_F | GTTTTGCTGGCGACGACG | qPCR |
| q_act1_R | ATACCACGCTTGCTTTGAG | qPCR |
| Tj1A | GTTTGCCGCACTTCAACCCGAAA | Northern blot probe |
| Tj1B | CGCGTTCTTCTGTCAACTCCTGCG | Northern blot probe |
| Tj1C | TGAGGCCGTTCAGTCATTCTTGC | Northern blot probe |
| Tj2A | ACCGTTGTTCTTCGTTATCCTTA | Northern blot probe |
| Tj2B | CCGTCCTCGTCTCCGTGCCA | Northern blot probe |
| Tj2C | CCGTCCTGTTCGTTCGAAAGAA | Northern blot probe |
| Tj3A | TCAGGTGTTCGTCTTCCGTCTCTG | Northern blot probe |
| Tj3B | TAACTCGTTCAGCTGTCGGTCCA | Northern blot probe |
| Tj3C | TTGAACCGTCCACATCGCCGTA | Northern blot probe |
| Tj4A | ATGCCTTCTCCACATTCCGGACA | Northern blot probe |
| Tj4B | GCGTTCTGTATTGTTCTTCATA | Northern blot probe |
| Tj4C | CTCGTCTTGTTCCGTCCGACATGT | Northern blot probe |
| Tj5A | TGGCGCTCCAGTTCTGTTCGTTCG | Northern blot probe |
| Tj5B | TTTGCGTTTTCGCTCGCGTCCGTA | Northern blot probe |
| Tj5C | CAGCTGTCGTCGTCCTCCGTGTA | Northern blot probe |
| Tj6A | CTCCATTGTTCTTTCTTCCGCTT | Northern blot probe |
| Tj6B | TCTCCAAAGCCGTCTGCCCGCAA | Northern blot probe |
| Tj6C | GATACCACTGCTTCGCGTCCATGT | Northern blot probe |
| Tj7A | CGCTTGCTTGCTCCACGTCCGCG | Northern blot probe |
| Tj7B | TGATGACAAGAACGACGGCGTCACC | Northern blot probe |
| Tj7C | GATCGTTCTCTCGCTCTGCCCATC | Northern blot probe |
| Tj8A | CGCCGCGCCCAGCCCTACGTCA | Northern blot probe |
| Tj8B | CATGTCAGTCGTTCTTCGTCGTCGA | Northern blot probe |
| Tj8C | ACTACCGTCCTTCTTCCTCACGAA | Northern blot probe |
| Tj9A | TACCGCTCCACTGTGCCCGAGTC | Northern blot probe |
| Tj9B | ATCGCGTCTTCCAATCCTCGTT | Northern blot probe |
| Tj9C | ATCGTCCGTTCAGACTGTCCGT | Northern blot probe |
| Tj10A | CACCGTTTTGTCCAGATCTTCA | Northern blot probe |
| Tj10B | TCGCTCCACTTGCATTTGTCCG | Northern blot probe |
| Tj10C | GCTTTGCTCGTCTAGTCCGCAA | Northern blot probe |
| jap_snoRNA58 | CTGCTAAATCAGAAGTCTAGCATC | Northern blot probe |
