# Supplementary material for: Separable roles for RNAi in regulation of transposable elements and viability in the fission yeast Schizosaccharomyces japonicus
Source: PLoS Genet. 2022 Feb 28;18(2):e1010100. doi: 10.1371/journal.pgen.1010100 (PMC8912903; doi:10.1371/journal.pgen.1010100)
Supplement: Attachment — Submitted filename: Response to Reviewers PLOSGenetics 250122.docx [file pgen.1010100.s017.docx]

**Response to Reviewers, manuscript PGENETICS-D-21-01431**

We thank the reviewers for their positive comments and constructive suggestions on our manuscript. In the revised version we believe we have addressed all of the points raised - a point-by-point response to the reviewers’ comments is provided below (our responses are highlighted in blue).

Reviewer #1: The study reported the study of the RNA interference pathway in Schizosaccharomyces japonicus. This yeast belongs to the fission yeast clade of which S. pombe is the most studied member. The authors report that the RNAi pathway is essential in J. japonicus in contrast to the situation in S. pombe. Still, they were able to identify rare viable dcr1delta transformants. In this background the cells do no produce siRNA and the level of expression of retrotransposon is increased so is the transposition of a least one them. In contrast, the authors observed no modification of the H3K9me2 marks at these retrotransposon loci. The analysis of genome of two of these rare dcr1dleta mutant revealed that they are suppressors bearing a mutation at the MPE1 and LEO1 locus, respectively. Overall, this study reveals the dual role of RNAi in S. japonicus controlling both the chromatin structure and the TE expression through a post-trancriptional pathway. This is well done study and a well written manuscript. Yet, several points need to be clarified as explained below:

1) Fig1B: the Northern indeed revealed that absence of TJ10 small RNAs but might suggest that it is not the case for TJ7. It is the RNA-Seq that revealed that the signal observed in the TJ7 Northern are not siRNA. So the sentence saying “Northern blot analysis indicated that retro-transposon-derived siRNAss were lost…” is false and should be modified.

We agree with this point and have modified the text accordingly: *“Northern blot analysis indicated that retrotransposon-derived small RNA populations were lost or altered...”*

2) The authors reported the identification of dcr1delta suppressor mutant but not ago1delta equivalent. Although they deleted AGO1 in the leo1 background. It is possible to do in the dcr1delta* mutant bearing the MPE1 mutation?

We agree that it would have been interesting to also test *ago1* deletion in the presence of the identified *mpe1* mutation, but as *mpe1^+^* is an essential gene, and as CRISPR-Cas9 approaches have not yet been developed for *S. japonicus*, we were unable to readily recreate the necessary point mutation in this gene.

3) The authors revealed an increased in the number of copies of TJ7 in the drc1* mutant. Where are these copies added in the genome? They did not observed any increase in the copy number of TJ10. Why these TJ10 retrotransposons do not transpose? Are they nonfunctional or truncated in some ways?

We do not know where the ‘new’ copies of Tj7 are integrated in the *dcr1∆** mutant. However, we note that Tj7 elements contain a chromodomain, a common feature of Ty3 type elements that has been shown to direct their integration into heterochromatic loci (Levin and Moran 2011, PMID: 21850042). We therefore suspect that additional copies of Tj7 will have integrated into existing heterochromatic TE arrays (making pinpointing of their exact locations difficult).

As to why Tj7 elements transpose in the *dcr1∆** mutant but Tj10 and others do not, we have looked into this, but do not currently have an explanation: we can find what appear to be full-length copies of most TEs including Tj10, so their failure to mobilise is not because they are truncated, and conversely there are no additional features in the sequence of Tj7 that could account for why this family alone is competent for transposition.

We have added discussion of both of these points in a new paragraph in the discussion.

4) What is the level of expression of TE in the leo1 background?

Levels of TE expression in the *leo1∆* background are very low, equivalent to those in wild-type cells. Although data on *leo1∆* cells was included in the original manuscript, we thank the reviewer for drawing our attention to the fact that it was not well displayed on the scale on which the data were originally plotted. We have therefore split the data in original Figure 4D into two separate plots (new Figures 4C and 4D) so that it is now clear that deletion of *leo1^+^* alone has little effect on TE expression.

5) According to the presented model, in the dcr1* mutant the only post-transcriptional repression pathway controlling TE expression is affected but still the expression of TJ7 increased by 200 fold in the presence of “normal” H3K9me2 marks. Could the authors comment on this apparent very poor efficiency of H3K9me2 marks to control gene expression in this yeast?

The levels of transcript accumulation for elements such as Tj7 in the *dcr1∆** cells are perhaps somewhat surprising given that these elements are nevertheless associated with H3K9 methylation. However it might be that not all copies of Tj7 are equally enriched for H3K9me2, and some are efficiently repressed while others are expressed (the high level of sequence identity means that it is not possible to distinguish between individual elements in ChIP/expression data). It could also be that this form of heterochromatin is permissive to transcription (as has been reported for H3K9me2 marked chromatin in *S. pombe*; Jih et al 2017, PMID: 28682306).

We have included discussion of this point in the new paragraph in the discussion.

Reviewer #2: It is well established that RNAi is a conserved mechanism that can suppress transposition of transposable elements (TEs) via transcriptional and post-transcriptional mechanisms. Interestingly, loss of RNAi in the model fission yeast Schizosaccharomyces pombe does not result in major defects in TE silencing, as the exosome RNA degradation machinery rapidly degrades transcripts from those elements to prevent their reactivation. In this manuscript, Chapman et al. probe the contribution of RNAi to TE regulation in Schizosaccharomyces japonicus, an organism that is closely related to S. pombe. Surprisingly, the authors discover and comprehensively demonstrate that RNAi and heterochromatin are essential for viability in S. japonicus, in contrast to what has been found for S. pombe. Importantly, the mutation or loss of Leo1, a subunit of the Paf1 complex, allows RNAi mutants to survive. H3K9me2 levels at TEs in such strains are unaffected but reactivation can occur, as some TEs are upregulated and one of them (i.e. Tj7) can transpose. In summary, the authors propose that RNAi regulates TEs in S. japonicus by two mechanisms, a transcriptional one involving heterochromatin formation and a post-transcriptional one involving degradation. Overall, the manuscript is well written and convincing. I would recommend it for publication in PLoS Genetics provided the following modifications are included:

Introduction/Discussion:
As mentioned, the authors must acknowledge that RNAi indeed plays an important role in controlling retrotransposons in S. pombe, but such a role is masked by the exosome RNA degradation machinery (Yamanaka et al., Nature 2012 PMID: 23151475). This raises the intriguing possibility that the exosome may not be targeting retrotransposons in S. japonicus, therefore rendering the role of RNAi essential.

We have clarified this point in the introduction (the reference to Yamanaka et al was already included): “TE transcripts are also subject to elimination by the exosome, and only in certain conditions, such as when exosome-mediated degradation is impaired, are TE transcripts strongly targeted by RNAi generating siRNAs that direct heterochromatin assembly at TE sequences”.

Figure 3A, ChIP-seq:
Please add the genome-wide data as a Supp. Figure, so the reader can judge the enrichment at heterochromatic regions in comparison to euchromatin.

We have added the genome-wide ChIP-seq data in new Figure S6.

Figs. 3B-C and S6, ChIP-qPCR:
While it is very clear that there is no difference in the enrichment levels between WT and various dcr1∆ mutants, the absence of negative controls makes it difficult to assess the quality of the ChIPs. Please show the raw unprocessed data as a Supp. Figure or Supp. Table.

To address this we have added negative control data for these ChIP-qPCRs (these controls had previously been done but not presented). The *act1^+^* locus is now shown as a negative control for H3K9me2 ChIP (Figure S7A, and for consistency also Figure S9A), and a no antibody control is shown for RNAPII ChIP (Figure S7B; the expressed *his3^+^* locus is also shown as a positive control). In each case all data have been replotted without normalisation to wild-type to enable evaluation of enrichment relative to the controls.

Figure 2D:
I wonder what it is the biological consequence of having a higher number of Tj7 copies and increased transcription of various Tjs in RNAi mutants. Could the authors provide serial dilution assays or liquid growth curves to assess the fitness of those strains (i.e. rare survivors and leo1∆ RNAi∆ double mutants)?

Indeed these strains with increased TE expression/activity display moderate growth defects, with growth phenotypes very similar between the rare survivors and the reconstituted *leo1∆ RNAi∆* double mutants. We have added a serial dilution assay showing this in Figure S10.

Uniqueness of Tj7
Is there any unique motif/s in the Tj7 sequence that might explain why this TE, but not other elements, is able to transpose?

We have looked into this but no, we have not been able to identify any unique sequence motif(s) in Tj7 that could account for why this element alone is competent for transposition. We have added a comment on this in the discussion.

Figure S5:
The authors state “Phylogenetic analyses indicated that each of the new TEs falls into one of the two existing retrotransposon lineages in S. japonicus (Figure S5)”. However, Tj19 is not displayed in Figure S5. This must be clarified. Please also state in the figure legend that Tj12-21 elements were identified in this study, whereas Tj11 was already known.

Identified Tj19 sequences were limited to only solo-LTRs and conserved gag-proximal zinc finger motifs; as the domains used for the phylogenetic analysis were not present, this element was excluded. We have clarified this point in the legend for figure S5: *“Tj19 was excluded as we could find only partial elements in which these domains were not present.”*

We have also clarified here the fact that Tj12-21 elements were identified in this study, whereas Tj11 was already known: *“Retrotransposons discovered in this study (Tj12 – Tj21) are highlighted in blue, previously discovered retrotransposons (Tj1 – Tj11) are highlighted in red.”*

The description about the existence of RNAi-independent heterochromatin assembly pathway shall include a reference to article by Reyes-Turcu et al (PMID: 21892171). Similarly, reference to Vo et al 2019 (PMID: 31269446) shall be included (along with ref 36) when describing the requirement of CPF complex in RNAi-independent heterochromatin assembly

These references have now been included.

Reviewer #3: Chapman and colleagues explore the role of RNAi in the fission yeast S. japonicus. This work is interesting on several grounds. As the authors highlight, RNAi has been investigated in the more intensely studied fission yeast S. pombe. The lineage leading to S. pombe has, however, acquired an RNAi-independent transposon defense pathway (not found in S. japonicus) and no longer relies heavily on RNAi for this role. S. japonicus, therefore, has a lot of potential as a model for studying RNAi. S. japonicus retains much of the tractability of S. pombe and the authors demonstrate that the S. japonicus RNAi pathway retains a role more like the ancestral state of the pathway shared with many other eukaryotes. I thought the paper was clear, accessible and the claims were mostly supported. I enjoyed reading this.

My one hesitation to feel the claims were fully reported was that the role for leo1 in S. japonicus has not been demonstrated. I agree with the authors that it is most likely the gene does the same thing in S. japonicus as it does in S. pombe. However, given the significant differences between the two species demonstrated in this work and the long time since they shared a common ancestor, it would be nice to have more support for the idea that Leo1 antagonizes H3K9 methylation in S. japonicus. The persistent inability to get clr4 KO even in a leo1 background helps convince me that the authors’ interpretations are correct, but figure 4B is not supported by a table with the number of transformants screened. Those data are provided for Figure 1A and I found that important.

We thank the reviewer for spotting this omission – in line with Figure 1A, the corresponding data for Figure 4B on the number of transformants screened has now been added to Figure S1.

I have some additional suggestions listed below. These are not major deficiencies but are mostly suggestions I think could help improve the paper.

Overall, I think more details about the japonicus transposons are warranted. I suggest adding more to introduce the transposon landscape in japonicus. How much more transposon dense is japonicus than pombe? What types of transposons are there (e.g. TF-like, Ty-like)? This was answered in the results, but I was wondering about this earlier in the paper. I was also confused by the naming system of japonicus transposons. I suggest the authors describe what determines if a transposon sequence represents a new family rather than a member of a previously defined family. Is there a formal cutoff of divergence that separates families? I was also wondering if there was any information about the mobility of the different transposon families, given they did not respond the same way to the mutations assayed in this paper.

As suggested, we have added additional background information on the transposon landscape in *S. japonicus* to the introduction. Tj1 to Tj11 family elements were identified previously, and we continued this naming convention for the additional families discovered here. Elements considered members of the same family had sequence identity in excess of 95% within the core domains (reverse transcriptase, RNase H and integrase), while between families maximum identity was 79%. This has now been more fully described in the methods.

Currently there is no further information about the mobility of the different transposon families, and we have not been able to identify anything in the sequences of the different elements that could account for why only Tj7 appears competent for transposition in the *dcr1∆** background. We have added discussion of this in a new paragraph in the discussion.

Were all assays done for the two dcr1 deletion mutants? The text says the phenotypes were indistinguishable, but that claim was hard to evaluate. Could those data be provided as a supplement?

A subset of assays were done for the second *dcr1∆* strain: the data are now shown in Figure S10.

Some more contextual information to introduce the motivation and expectations in the PolIII CHiP qPCR could help general audience readers.

As suggested we have added some additional text explaining this.

In the discussion, the term ‘higher eukaryotes’ was used. I suggest rewording to multicellular eukaryotes.

We agree that it is preferable to avoid this expression and have changed it as suggested.

The centromeric cohesin/chromosome segregation model for the H3K9 methylation requirement seems reasonable. Is it also possible that too much transposon activity could contribute to the lethality associated with loss of H3K9 methylation?

We felt that excessive transposon activity was less likely to be a major contributor to the lethality associated with loss of H3K9 methylation since TE dysregulation was also seen in the *leo1∆ dcr1∆* background where H3K9 methylation was maintained and viability restored. However, it is possible that transposon-related damage may be further exacerbated in the absence of H3K9 methylation, and therefore could also contribute to lethality. We have now included mention of this in the discussion.

Figure 3A. Please provide more info about the scale.

A description of the scale has been added to the legend of Figures 3 (and corresponding new Figure S6).

Figure S1: There are several genes not mentioned in the paper. Folks may miss them.

Agreed – we now include mention of these other genes in the main text.

For some of the experiments, I think the same experiments shown in the main text and supplemental figure. I appreciate that as a reader as it makes comparisons easier. I suggest, however, mentioning in the legend of the supplement if this is the same data shown again.

In two of our supplementary figures (S4 and S9) we have indeed included data points also shown in the main figures for ease of comparison. Where this has been done we have now clearly stated this in the corresponding figure legend.

For Figure S5, what are the ‘three loci’? There seem to be many more than three considered in the tree.

Here we were referring to the three TE domains used to construct the phylogeny: the reverse transcriptase, RNase and integrase domains. We realise now that this was confusing, and therefore have removed the reference to ‘three loci’ from the figure title, and have replaced ‘loci’ with ‘domains’ in the body of the legend.

Figure S8: The letters are missing from the figure.

We thank the reviewer for spotting this error – it has now been corrected.
